# Fluorescence-Activated Cell Sorting Depletes Macrophages and Triggers Inflammation in the Single-Cell Immune Landscape of Human Atherosclerosis

**DOI:** 10.1161/ATVBAHA.125.322856

**Published:** 2025-06-05

**Authors:** Ayden G. Case, James W. O’Brien, Fiona T.W. Charlier (唐婺盈), Ali B.A.K. Al-Hadithi, Mohammed M. Chowdhury, Stephen A. Newland, Zixuan Huang (黄梓煊), Gemma Basatemur, Xiaohui Zhao, Ayoola I. Awopetu, Jonathan R. Boyle, Nicholas R. Evans, Ziad Mallat, Tian X. Zhao

**Affiliations:** 1 Department of Medicine (A.G.C., J.W.O., F.T.W.C., A.B.A.K.A.-H., S.A.N., Z.H., G.B., X.Z., Z.M., T.X.Z.), University of Cambridge, United Kingdom.; 2 Department of Surgery (J.R.B.), University of Cambridge, United Kingdom.; 3 Department of Clinical Neurosciences (N.R.E.), University of Cambridge, United Kingdom.; 4 National Heart, Lung, and Blood Institute, National Institutes of Health (NIH), Complement and Inflammation Research Section, Bethesda, MD (A.G.C.).; 5 Department of Surgery, Cambridge University Hospital National Health Service (NHS) Trust, United Kingdom (M.M.C., A.I.A., J.R.B.).; 6 Université Paris Cité, Institut National de la Santé et de la Recherche Médicale, France (Z.M.).; 7 Department of Cardiology, Royal Papworth NHS Foundation Trust, United Kingdom (T.X.Z.).

**Keywords:** atherosclerosis, immunity, inflammation, macrophages

Atherosclerosis is characterized by a maladaptive immune response. Our understanding of this immune process has been advanced by using single-cell RNA sequencing (scRNA-seq) performed on human carotid plaques. The most widely used scRNA-seq platform utilizes a droplet-based microfluidics workflow (10× Genomics) and requires a debris-free single-cell suspension for optimal performance. Accordingly, most investigators studying human atherosclerosis use fluorescence-activated cell sorting (FACS) after tissue digestion.^1–4^ However, the impact of FACS on the downstream scRNA-seq workflow and the immune cells profiled remains unknown. SPLiT-seq (Split-pool ligation-based transcriptome sequencing), a recently developed scRNA-seq technique, is more tolerant of debris and therefore offers a unique opportunity to assess the effect of FACS compared with CD45^+^ magnetic bead enrichment on the single-cell immune landscape of human atherosclerosis.

Human carotid plaques were obtained as part of a clinical study: Wales Research Ethics Committee, United Kingdom (22/WA/0013; https://www.clinicaltrials.gov; Unique identifier: NCT05975554). The methodology is outlined in the Figure (A). Each plaque was washed, minced, and then digested in 450 U/mL collagenase I, 125 U/mL collagenase XI, 60 U/mL hyaluronidase, elastase suspension (20 µL/mL), and 60 U/mL DNAse I. An agitator at 37 °C was used for 75 minutes, then quenched using complete media (RPMI [Roswell Park Memorial Institute]+10% FBS). This was passed through a 70 µm filter and then centrifuged (400*g*, 6 minutes) and resuspended in complete media. The suspension was then split equally into 2: (1) FACS and (2) bead enrichment.

**Figure.**
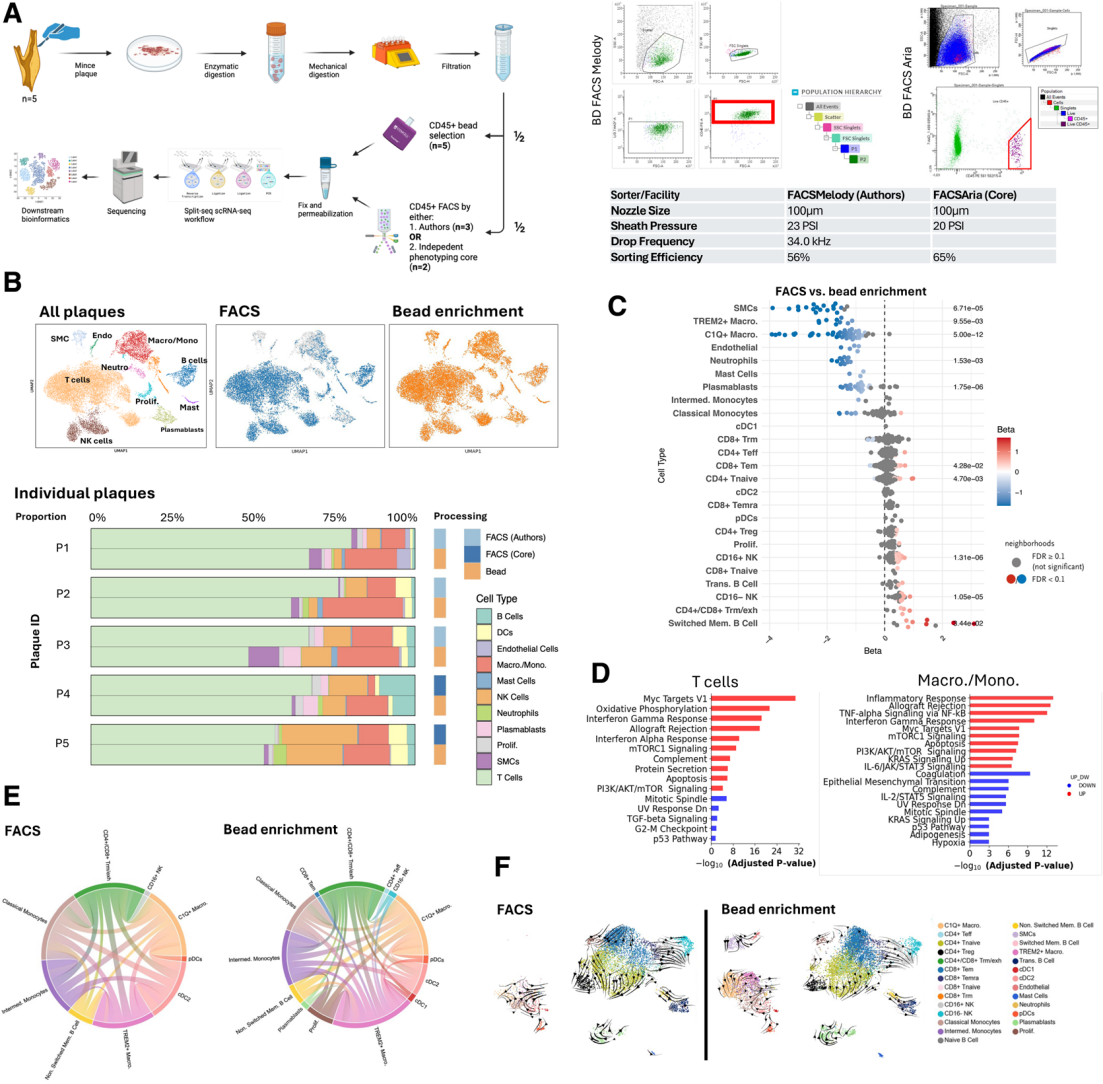
**Fluorescence-activated cell sorting (FACS) used in single-cell RNA sequencing workflows depletes macrophages and triggers inflammation. A**, Overview of experimental design (left; made with biorender.com). Gating strategy for the 2 fluorescence-activated cell sorting (FACS) setups used including sorting parameters (right). **B**, Uniform Manifold Approximation and Projection (UMAP) shows coarse cell groups from all plaques while middle and right UMAPs show cells recovered from FACS and bead enrichment projected onto the UMAP. Stacked bar chart showing proportion of cell types recovered from individual plaques split by sorting method. FACS was performed by the authors or an independent phenotyping hub. **C**, Differential abundance testing of single-cell neighborhoods between FACS and bead enrichment groups with a negative binomial generalized linear mixed-effects model of all plaques. The plot shows the beta coefficients for each neighborhood assigned to corresponding cell types where positive and negative coefficient values are interpreted as enriched or depleted, respectively, in the FACS condition. Differentially abundant neighborhoods are colored according to the beta coefficient value from blue to white to red, where white indicates a value of 0 (no change). Nonsignificant neighborhoods are colored gray. *P* value is a single sample Wilcoxon *u*=0 for any neighborhood with spatial FDR <0.1 for that specific sample, with Benjamini-Hochberg FDR correction. **D**, Pathway analysis using Hallmark. Red and blue bars represent the upregulation and downregulation of pathways, respectively, by comparing FACS to bead enrichment. **E**, Circos plot showing receptor-ligand interaction analysis. Thickness of the connections represents the proportion of interactions. **F**, RNA velocity analysis where the arrows on the UMAP indicate the directions of the predicted future transcriptional states of the cells. C1Q indicates complement component 1q; cDC, conventional dendritic cells; Endo, endothelial cells; FDR, false discovery rate; FSC, forward scatter; Intermed., intermediate; Macro/Mono, macrophages/monocytes; Mem., memory; Neutro, neutrophils; NK, natural killer; pDC, plasmacytoid dendritic cells; Prolif., proliferating; PSI, pounds per square inch; SMC, smooth muscle cells; scRNA-seq, single-cell RNA sequencing; SSC, side scatter; Teff, effector T cells; Tem, effector memory T cells; Temra, terminally differentiated effector memory T cells; Trans., transitional; Treg, regulatory T cells; TREM2, triggering receptor expressed on myeloid cells; and Trm, tissue-resident memory T cells.

FACS cells underwent staining for CD45 (PE [phycoerythrin]) and live/dead (7AAD [7-aminoactinomycin D]). The stained solution was then sorted for live, CD45^+^ cells either by the authors using a BD FACSMelody or by an independent core immunophenotyping facility using a BD FACSAria Fusion. FACS cells were collected in complete media supplemented with an additional 10% FBS and then placed on ice. The duration of FACS varied but averaged 45 minutes.

Bead-enriched cells underwent CD45^+^ selection using the EasySep CD45^+^-positive selection kit. Cells were resuspended in PBS supplemented with 10% FBS and 1% EDTA. Polystyrene round bottom tubes were used. The duration of CD45^+^-positive magnetic bead selection was ≈70 minutes.

Both bead-enriched and FACS-treated conditions were then immediately fixed using the Evercode Cell Fixation Kit v3 (Parse Biosciences; in LoBind tubes blocked with 1% bovine serum albumin) and subsequently underwent library preparation (Evercode WT [Whole Transcriptome] v3–v1.5). Libraries were sequenced on the Illumina platform. A standard downstream bioinformatics pipeline used Scanpy with Harmony integration. Differential abundance analysis used Pertpy’s (v0.9.50) implementation of Milo. Gene Set Enrichment Analysis used Gseapy (v1.1.4). Receptor-ligand interaction used CellPhoneDB (v5.0.1). RNA velocity used ScVelo (v0.3.3). Code available at https://github.com/zhaolabcambridge/SortedPlaqueAnalysis. The data that support the findings of this study are available from the corresponding author upon reasonable request.

Between April and November 2024, 5 patients undergoing carotid endarterectomy surgery were recruited. From the 5 plaques, 20 293 single cells passed quality control. When assessing coarse cell types across all 5 plaques, FACS causes marked depletion of macrophages/monocytes and plasmablast cell types (Figure [B]). When assessing proportions, FACS results in a mean plaque composition of 67% T cells, 13% natural killer cells, 7% B cells, 5% macrophages/monocytes, and 2.6% dendritic cells. In contrast, CD45^+^ bead selection results in a mean plaque composition of 60% T cells, 8.5% natural killer cells, 4.1% B cells, 15% macrophages/monocytes, and 2.2% dendritic cells. FACS resulted in purer CD45^+^ cell suspensions with fewer vascular smooth muscle and endothelial cells (2.5% versus 0.2% and 0.9% versus 0.2%, respectively). This pattern is seen consistently across all 5 plaques and in FACS performed by either the authors or independent core immunophenotyping facility (Figure [B]). Using differential abundance analysis, we show a statistically significant depletion of smooth muscle cells, TREM2+ and C1Q+ macrophages, neutrophils, and plasmablasts using FACS. This is likely due to macrophages being larger, more fragile, and more adherent to plastics, causing size exclusion and stress-induced apoptosis during sorting. Conversely, switched memory B cells, exhausted T cells, natural killer cells, CD4+ naive T cells, and CD8+ effector memory T cells were marginally increased with FACS (Figure [C]).

We next examined the effect of FACS compared with bead selection on the single-cell transcriptome across the 2 largest coarse cell types, T cells, and macrophages/monocytes. Pathway analysis showed significant upregulation of gene sets associated with inflammation (eg, allograft rejection, interferon-gamma response, Myc targets V1) in both cell groups in the FACS condition (Figure [D]). In addition, FACS caused upregulation of genes (*HSPA1A, HSP90AA1, HIF1A, XBP1*, and *NFKBIA*) associated with a stress response in T cells.

Cell-cell communication inference by ligand-receptor interaction analysis showed that FACS resulted in fewer communications. To determine whether this effect was driven by changes in cell proportions or alterations in cell transcriptomics, we randomly down-sampled immune cell numbers to equalize cell type proportions across conditions and confirmed the result (Figure [E]). RNA velocity analysis showed similar trajectories (going from proliferating cells to CD8 T effector memory cells, naive T cells to regulatory T cells, and nonswitched memory B cells to switched memory B cells) between the 2 conditions (Figure [F]).

In conclusion, although the effect of FACS on various immune cell types has been studied,^5^ this study uniquely examines the impact of FACS on scRNA-seq outcomes. In many circumstances, FACS and bead enrichment have different downstream applications. When studying human atherosclerosis, we show that FACS is more effective at producing a cleaner single-cell suspension, characterized by a higher proportion of CD45^+^ cells. However, with our samples, this comes at the cost of depleting macrophages and inducing an inflammatory shift in the single-cell transcriptome. We have not investigated the optimal pre-FACS treatment or FACS parameters but instead have adapted published workflows used in human atherosclerosis,^1–4^ therefore limiting the generalisability of these findings to FACS used in other scenarios. Indeed, additional FACS optimization, such as debris removal, may improve downstream scRNA-seq performance and warrants future investigation. In conclusion, these novel findings provide critical insights and considerations for optimizing scRNA-seq workflows in studies of complex human disease tissues.

## Article Information

### Acknowledgments

The authors acknowledge the Department of Vascular Surgery and Department of Stroke at Cambridge University of Hospital NHS Trust for their support in this study.

### Sources of Funding

A.G. Case acknowledges funding from the Gates Cambridge Trust and the National Institutes of Health Oxford-Cambridge Scholars Program. This work was supported by the British Heart Foundation (T.X. Zhao: IA/F/23/275046, FS/ICRF/24/26114. Z. Mallat: CH/10/001/27642, RE/24/130011, RE/18/1/34212, PG/23/11508, and J. O’Brien: FS/CRTF/24/24605), Heart Research UK (RG2699/21/23, T.X. Zhao and Z. Mallat), and the British Medical Association (T.X. Zhao).

### Disclosures

None.
